# Cognitive Neurology and Ageing Scientic Department of the Brazilian Academy of Neurology

**Published:** 2008

**Authors:** Paulo Caramelli, Márcia Lorena Fagundes Chaves, Rogério Gomes Beato

## Biennial report – 2006-2008

Our two-year term as coordinators of the Cognitive Neurology and Ageing Scientific Department (CNASC) of the Brazilian Academy of Neurology has come to an end this August. We now take this opportunity to highlight some activities conducted during this period.

1. Implementation of the CNASC web page hosted on the Brazilian Academy of Neurology site (www.abneuro.org). This web page contains a range of content and information which may be useful to our colleagues, namely:
  - A brief historical appraisal of the Department;
  - Brief information for the lay public about Cognitive Neurology;
  - A list of services and research groups on Cognitive Neurology and Ageing in Brazil coordinated by members of the Brazilian Academy of Neurology who belong to the CNASC;
  - A survey on cognitive tests adopted by the main services of Cognitive Neurology and Ageing in different parts of the country, with special focus on the Mini-Mental State Examination;
  - A list of some Brazilian studies published in the area of dementia;
  - Articles in PDF produced by the CNASCand published in Arquivos de Neuro-Psiquiatria, the official journal of the Brazilian Academy of Neurology: Consensus recommendations on diagnosis (parts 1 and 2) and treatment of Alzheimer disease in Brazil; a glossary of frequent terms used in Cognitive and Behavioral Neurosciences translated into Portuguese;
  - Important information on prion diseases, including the forms required for compulsory notification of Creutzfeldt-Jakob disease;
  - Instructions for authors of **Dementia & Neuropsychologia**, the official journal of the CNASC;
  - Articles in PDF format (open access) published in **Dementia & Neuropsychologia**, since its first issue (March, 2007).
Regular contributions to NeuroAtual, an official publication of the Brazilian Academy of Neurology (six issues per year) which presents comments on recently published original and review articles of interest to the neurologist. Besides the important role of NeuroAtual in continuous medical education for the general neurologist, the participation of the CNASC aimed to call the attention of our colleagues, including residents, to the fascinating field of Cognitive Neurology.
Participation in a meeting on Attention Deficit Hyperactivity Disorder (ADHD) held in Rio de Janeiro, in November, 2007, by the Brazilian Association of Attention Deficit. This meeting was co-sponsored by different scientific associations, including the Brazilian Academy of Neurology. The CNASC was represented by our colleague Wellington Borges Leite. We believe that the inclusion of ADHD, albeit in children, adolescents and adults, into the agenda of our Department is an important step to reinforce the role of the neurologist in the diagnosis and treatment of this disorder.
Organization of the VI Brazilian Meeting of Researchers in Alzheimer Disease and Related Disorders, held in Ouro Preto, Minas Gerais state, from December 6th to 8th, 2007. This is the main scientific activity of the CNASC, organized biennially since 1997, and constitutes the most important event in the area of dementia research in Brazil. As usual, no registration fee was charged to the conveners and their participation was based on the approval of an oral or poster presentation.
  The 2007 edition saw a record number of submitted abstracts: 235, representing a 45% increase in relation to the previous meeting. Of this total, some 210 were selected for presentation. Specific awards were granted for the best presentations on basic and clinical research. All the abstracts were published in a special supplement of **Dementia & Neuropsychologia**.

During the XXIII Brazilian Congress of Neurology, held last August in Belém, Pará state, a new board of coordinators was elected to lead the CNASC up until 2010.

Prof. Márcia Lorena Fagundes Chaves, from the Federal University of Rio Grande do Sul, in Porto Alegre, who worked as vice-coordinator from 2006 to 2008, is now the new coordinator. Dr. Ivan Hideyo Okamoto (from the Federal University of São Paulo) and Dr. Cláudia da Cunha Godinho (also from the Federal University of Rio Grande do Sul) were elected as vice-coordinator and secretary, respectively. They will certainly work hard to best represent all members of the CNASC and to significantly expand our activities.

We wish to thank all our friends and colleagues from the CNASC who kindly supported us during this two-year term.

**Paulo Caramelli** COORDINATOR**Márcia Lorena Fagundes Chaves** VICE-COORDINATOR**Rogério Gomes Beato** SECRETARY

